# Multiphase evaluation of portable medicines quality screening devices

**DOI:** 10.1371/journal.pntd.0009287

**Published:** 2021-09-30

**Authors:** Céline Caillet, Serena Vickers, Stephen Zambrzycki, Nantasit Luangasanatip, Vayouly Vidhamaly, Kem Boutsamay, Phonepasith Boupha, Yoel Lubell, Facundo M. Fernández, Paul N. Newton

**Affiliations:** 1 Lao-Oxford-Mahosot Hospital-Wellcome Trust Research Unit, Microbiology Laboratory, Mahosot Hospital, Vientiane, Lao PDR; 2 Centre for Tropical Medicine and Global Health, Nuffield Department of Medicine, University of Oxford, Oxford, United Kingdom; 3 Infectious Diseases Data Observatory (IDDO)/World Wide Antimalarial Resistance Network (WWARN), University of Oxford, Oxford, United Kingdom; 4 Mahidol Oxford Tropical Medicine Research Unit (MORU), Faculty of Tropical Medicine, Mahidol University, Bangkok, Thailand; 5 School of Chemistry and Biochemistry, Georgia Institute of Technology, Atlanta, Georgia, United States of America; George Washington University School of Medicine and Health Sciences, UNITED STATES

Substandard and falsified (SF) medicines have important but neglected consequences including increased morbidity and mortality, economic losses, and diminished public confidence in health systems. SF antimicrobials, particularly those containing reduced quantities of active pharmaceutical ingredients (APIs), may also be key but overlooked drivers of antimicrobial resistance [1]. Substandard medicines result from negligence and errors made during the manufacturing process by authorized manufacturers or degradation in supply chains. Falsified medicines are the result of criminal activity. Falsified medicines purport to be real, authorized medicines but are deliberately and fraudulently mislabeled with respect to their identity and/or source [2]. Falsified medicines usually have packaging that are copies of a genuine product and may contain the APIs, although often at the incorrect amount, or, more commonly, they contain other API(s) or none at all. The term “falsified medicines,” adopted by the World Health Assembly in May 2017, references the public health issues of poor quality medicines rather than the term “counterfeit” that refers to trademark infringement. As countermeasures vary according to the type of “defect,” understanding the differences between the types of poor quality medicines is essential from a public health and regulatory perspective.

According to a recent report from the World Health Organization (WHO), approximately 10% of medicines circulating in low- and middle-income countries (LMICs) are either substandard or falsified [3]. The issue appears to be of greater magnitude in LMICs than in wealthier countries [4–6].

The Coronavirus Disease 2019 (COVID-19) pandemic has heightened the risk for SF medicines reaching patients by disrupting pharmaceutical production and supply chains, impeding regulatory inspection, and causing great economic hardship, making it difficult to afford genuine medicines. A surge of SF medical product cases has been reported, especially in settings with already vulnerable supply chains [7–10]. In early April 2020, soon after chloroquine and its derivatives were widely publicized for their potential efficacy for COVID-19 treatment and/or prevention, WHO alerted about falsified versions of chloroquine in 5 countries, of which 4 were African LMICs [11]. The Medicine Quality Monitoring Globe identified Google News reports of 2 English language newspaper articles about COVID-19 medical products quality issues in January 2020, rising to 576 in February 2021 [9,12]. As new medical products are developed for COVID-19 treatments and prevention, it is highly likely that the incidence of SF products will increase in our stressed pharmaceutical world.

Medicines Regulatory Authorities (MRAs) are the keystones for many of the interventions to prevent, detect, and respond to SF medicines. However, national MRA, international procurement agencies, and wholesaler/distributor medicine inspectors performing post-marketing surveillance (PMS) have to largely rely only on their own senses and knowledge to detect circulating SF medicines [13]. Suspicious samples may be sent to formal chemical analysis laboratories for further advanced chromatographic assays such as high-performance liquid chromatography (HPLC) or liquid chromatography-mass spectrometry (LC-MS), but these assays are expensive, time-consuming, and not readily available in many countries. These complex assays and centralized laboratory testing lead to significant delay between collection of the suspicious medicine and confirmation of its poor quality, with its harm spreading unchecked in the interim. Rapid detection of SF medicines in the field is key to inform timely actions to prevent unsafe poor quality medicines from reaching patients. In 2018, the Member State Mechanism on SF medical products raised the improvement of detection as an important aspect in the fight against SF medicines at the World Health Assembly [14].

As an example of regulatory practice, in the Lao People’s Democratic Republic (Lao PDR, Laos), where much of the data were collected for the multiphase study described in this *PLOS Neglected Tropical Diseases* Collection, typically, inspection of medicines quality is conducted by medicine inspectors from the Bureau of Food and Drug Inspection (BFDI) within the Ministry of Health. Inspectors undertake routine inspection of pharmacies bi-annually, focusing on adherence to legislation (e.g., appropriate paperwork completion) and drug registration. Convenience sampling of certain medicines, especially antimalarials and antiretrovirals, is undertaken as part of specific vertical program projects supported by donors. In convenience sampling within a province, medicines are purchased from a selection of pharmacies in each district and brought back to a central location where they undergo initial screening using the Minilab [15]. All samples that fail Minilab screening conducted in regional offices and a further 10% of those that pass are then sent to the national Food and Drug Quality Control Center (FDQCC) for pharmacopeial testing.

Over the last 2 decades, a diversity of portable devices have been developed to better equip medicine inspectors to detect suspect medicines, offering the potential for more objective analysis of medicines in the “field” [16,17]. These devices have a great breadth in underlying technology, outputs, limitations, cost, training requirements, and ease of use. They range from sophisticated and expensive handheld Raman and near-infrared devices to a portable thin-layer chromatography kit to single-use paper cards and cassettes. This plethora of devices holds great hope for empowering medicine inspectors, making their work more actionable and cost-effective and improving MRA capacity to protect patients from harmful SF medicines. The need for such innovation is emphasized by the fragile character of pharmaceutical supply systems, as highlighted by the consequences of the COVID-19 pandemic. However, there are enormous key gaps in the scientific evidence to inform national MRA of the optimal, cost-effective choice of device to detect and respond to SF medicines [13,17]. This includes the lack of independent comparative evaluation of the majority of devices particularly in field settings, the amount and nature of training required for accurate use, an assessment of where in the supply chain different devices are best employed, and the cost-effectiveness of introducing devices within PMS systems. These gaps of knowledge impede decisions on how to best use these portable devices [13,17].

In this *PLOS* Collection, “A multiphase evaluation of portable screening devices to assess medicines quality for national Medicines Regulatory Authorities,” we describe the results of the multiphase collaborative study conducted between 2016 and 2018 that was part of the Results for Malaria Elimination and Communicable Diseases Control (RECAP) under the Regional Malaria and Communicable Disease Trust Fund (RMTF) at the Asian Development Bank. The study aimed to evaluate the accuracy, utility, usability, and cost-effectiveness of different portable devices to identify SF medicines across a variety of essential anti-infective medicines commonly used in the Greater Mekong Sub-region (GMS) to treat malaria and bacterial infections. This project was conducted in parallel with the United States Pharmacopeia Technology Review program [13,18,19].

**Fig 1:**
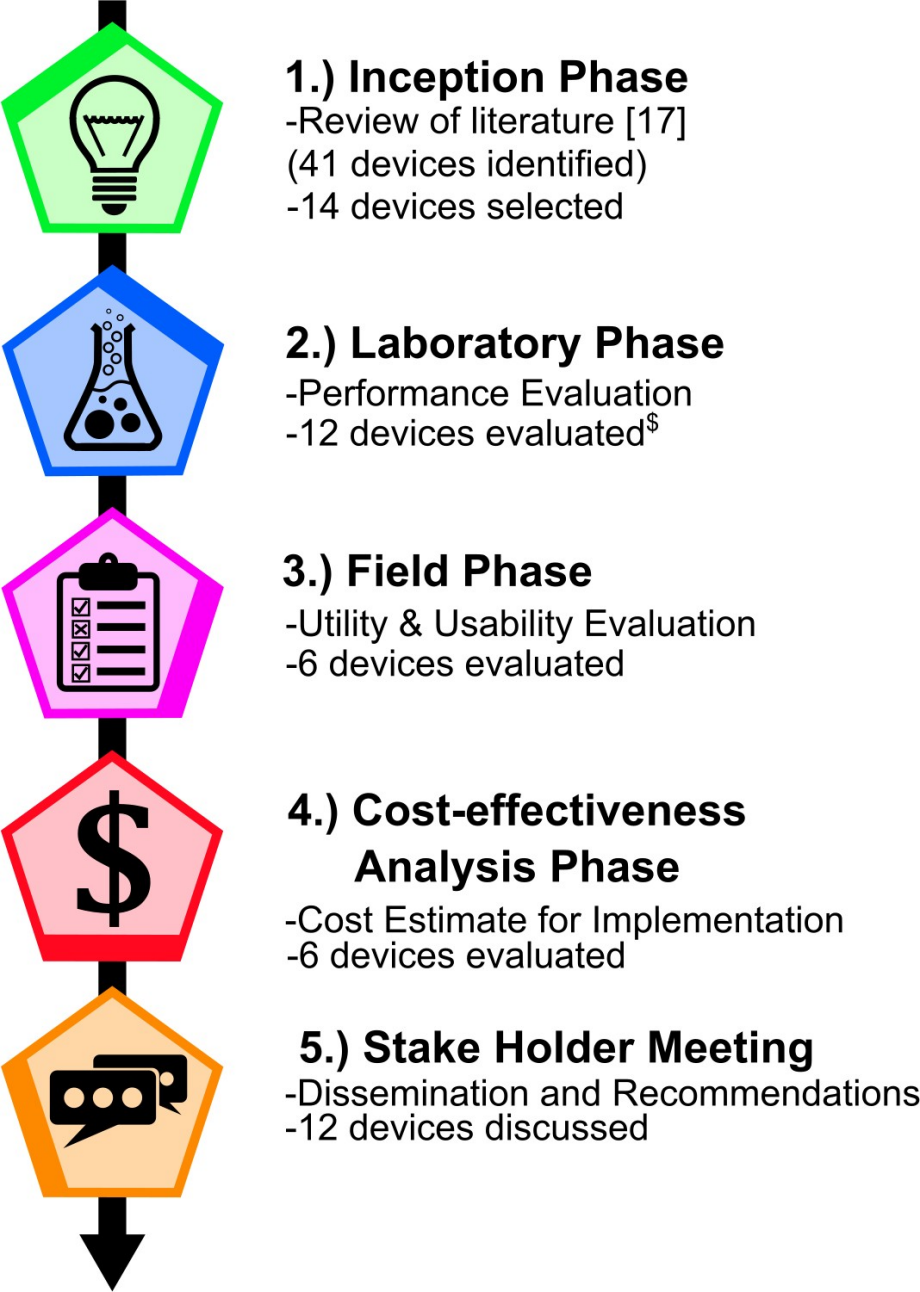
Diagram of the outline for the overall project. ^$^ The CoDI (developed by the United States CDC)—a device that uses laser absorption and fluorescence—could not be assessed because of intellectual property issues. A misunderstanding of the operational procedures of the CD3+—a device using UV-Vis-IR light to reveal differences in the packaging as compared to its genuine counterpart (developed by the US FDA)—led to potential significant bias in the performance results of the device during its evaluation. Those are thus not presented in this article. CDC, Centers for Disease Control and Prevention; CoDI, Counterfeit Detection Indicator; FDA, Food and Drug Administration.

The Collection is composed of 5 articles. This Editorial represents the first of these, introducing the background and rationale for the Collection. Twelve devices were first evaluated in a laboratory setting (“laboratory evaluation”) to provide information on their performances to identify SF medicines and to select the most field-suitable devices for further preliminary evaluation of their utility/usability (“field evaluation” phase) by medicines inspectors (**Fig 1**). Using part of the data gathered in the laboratory and the field evaluations, a cost-effectiveness evaluation [“cost-effectiveness analysis” (CEA)] of the implementation of the 6 devices tested in the field evaluation for PMS in Laos was conducted. The fifth article discusses the results presented in the series, highlights the evidence gaps, and provides recommendations on the key aspects to consider in the implementation of portable devices and their main advantages/limitations.

The laboratory evaluation included 12 devices selected from a literature review and expert advice [17]. Each device was evaluated on its ability to detect good and poor quality medicines, including field-collected samples (mainly from the GMS), and simulated SF samples produced in the laboratory. The devices’ performance, consumable needs, training requirements, and setup are reported. These results were utilized to select the most field-suitable devices for the field study.

In the field study, an evaluation pharmacy was constructed to resemble a Lao type 2 pharmacy to evaluate the utility and usability of devices. Inspectors were observed by members of the medicine quality research group while doing simulated pharmacy inspections. This allowed the recording of time taken to conduct evaluation pharmacy inspections with the devices and the observation of potential user errors when using the device in a real-life pharmacy setting. Inspectors were also observed while testing a predefined sample set of medicines (SSM) with the devices in an office setting, allowing more scrutiny by the observers. The GPHF-Minilab was also tested by the chemists in the laboratory evaluation, and by FDQCC technicians, already trained in Minilab use, at their laboratory, in line with its current routine use in Laos.

Using results of the above studies, the fourth article presents a CEA of implementing the 6 devices selected for the field phase experiments. Key criteria for each device such as cost per sample analysis, the sensitivity and specificity of each device, and experiment time were utilized to estimate the cost-effectiveness of using the devices under 2 scenarios of high and low prevalence of SF antimalarials in circulation. The results include a variety of device sampling strategies and multiway head-to-head comparisons.

In the fifth article, these 4 components are synthesized and discussed, and recommendations are made. This is based on the discussion in a multi-stakeholders meeting held in April 2018 in Vientiane. The meeting included 53 participants from MRAs from 7 Asian and African countries and international health and funding organizations. Hands-on sessions with devices and group discussions about the promises and pitfalls of the devices were held during this 2-day meeting. The advantages/disadvantages, cost-effectiveness, and optimal use of medicine quality screening devices in the medicine supply chains were discussed. Based on the results from this multiphase study, we list policy recommendations for MRAs and other institutions who wish to implement screening technologies, as well as gaps of scientific knowledge to be filled.

As far as we are aware, this multiphase study is the first collaborative independent investigation listing the advantages and disadvantages from diverse chemical, economical, and regulatory points of view. The overall study assesses and compares device accuracy, describes potential barriers, and evaluates the costs versus the benefits of the implementation of a wide diversity of portable medicine quality screening devices in a public health perspective. The series also highlights the difficulties and barriers to perform screening devices research and the important remaining gaps of scientific evidence.
